# Molecular Characterization and Epidemiology of Lumpy Skin Disease Virus in Bhutan, 2023

**DOI:** 10.1155/tbed/8634585

**Published:** 2025-12-11

**Authors:** Puspa M. Sharma, Pelden Wangchuk, Dawa Tshering, Tenzin La, Sangay Rinchen, Nirmal K. Thapa, Tirumala B. K. Settypalli, William G. Dundon, Irene K. Meki, Charles E. Lamien

**Affiliations:** ^1^ National Centre for Animal Health, Department of Livestock, Ministry of Agriculture and Livestock, Serbithang, Thimphu, Bhutan, moal.gov.bt; ^2^ Animal Production and Health Laboratory, Joint FAO/IAEA Centre of Nuclear Techniques in Food and Agriculture, Department of Nuclear Sciences and Applications, International Atomic Energy Agency, Wagramer Strasse 5 P.O. Box 100, Vienna, A-1400, Austria, iaea.org

**Keywords:** Bhutan, cattle, LSDV, outbreak, phylogenetic analysis, yak

## Abstract

Lumpy skin disease (LSD) is a disease of cattle and other ruminants caused by the LSD Virus (LSDV). LSD infections are characterized by high morbidity, low mortality, and significant economic impact. Initially endemic to Africa only, LSD has spread to the Middle East, Europe, and Asia in the past decade. In 2023, LSD posed a significant threat to Bhutan’s livestock population, with outbreaks reported across 20 Dzongkhags (districts) and 192 Gewogs (groups of villages). This study investigated the epidemiology and molecular aspects of the outbreak. A total of 19,907 animals (16,728 cattle and 3179 yaks) were affected, with 2888 deaths recorded, leading to an apparent case fatality rate (CFR) of 9.92% in cattle and 38.66% in yaks. Molecular characterization of the LSDV‐positive samples from cattle and yaks based on LSDV‐differentiating genes (i.e., RPO30, GPCR, EEV, and B22R) revealed 100% similarity among the samples, clustering them with LSDV field isolates from Sudan, India, and China in Clade 1.2. Further whole‐genome sequence characterization of a representative sample (LSDV_Bhutan_03) from cattle skin scrapings and phylogenetic network analysis clustered the virus with Neethling Warmbaths (NW)‐like LSDVs (Clade 1.2.2). Within the NW‐like clade, the Bhutan LSDV was closely related to recent isolates from cattle and buffalo in India and yaks in China. These data highlight the importance of LSDV surveillance in both domestic and wild bovines to identify spillover incidences, understand the extent of disease spread, and strengthen control measures.

## 1. Introduction

Lumpy skin disease virus (LSDV; species name *Capripoxvirus lumpyskinpox*), which causes lumpy skin disease (LSD), belongs to the genus *Capripoxviru*s, along with sheeppox virus (SPV; species name *Capripoxvirus sheeppox*) and goatpox virus (GPV; species name *Capripoxvirus goatpox*), within the subfamily *Chordopoxvirinae* of the family *Poxviridae* [1]. LSD affects cattle (*Bos taurus*) and other ruminants (for example zebus [*Bos taurus indicus*], buffalo [*Bison bison*], elands [*Taurotragus oryx*], impalas [*Aepyceros melampus*]), and is characterized by fever, nodules on the skin, mucous membranes and internal organs, emaciation, enlarged lymph nodes, and edema of the skin [2]. LSDV is economically significant as it can cause a temporary reduction in milk production, temporary or permanent sterility in bulls, damage to hides, and occasionally death. The World Organization for Animal Health (WOAH) categorized LSD as a notifiable disease due to its substantial economic losses and the international trade restrictions on live animals and their products [3, 4].

Historically, LSD was first reported in Zambia in 1929 and was confined to sub‐Saharan regions until 1986. The disease then spread to Middle Eastern countries and since 2019, countries such as India [5], China [6], Nepal [7], and Bangladesh [8] have reported their first cases of LSD. In anticipation of the potential spread of LSDV, Bhutan, located between India and China, has been strengthening preparedness against potential LSD incursion. This includes developing the National LSD Prevention and Control Plan, heightening surveillance and monitoring at southern border entry points, and the establishment of molecular detection assays for LSDV at the national laboratory.

On 5 October 2020, the National Centre for Animal Health (NCAH) confirmed the presence of LSDV DNA in cattle tissue samples from Samtse, a district in southwest Bhutan, using real‐time PCR. Following this, the virus spread to the southern regions of the country. During this period, Bhutan implemented frequent COVID‐19 lockdowns, which initially helped contain LSDV within the southern districts. However, after restrictions were lifted, LSDV cases surged, leading to widespread outbreaks in 2023. By the summer of 2023, the disease had spread to all districts, affecting both cattle and yaks (*Bos grunniens*).

While a previous study [9] explored the occurrence of LSDV in Bhutan, the molecular profile of LSDV strains circulating in the country remained unknown. To address this knowledge gap, this study investigated the epidemiology and molecular aspects of the 2023 LSD outbreak by characterizing LSDV strains from samples confirmed positive by qPCR at the NCAH in Thimphu from March to September 2023.

## 2. Materials and Methods

### 2.1. Outbreak Investigation and Sample Collection

The 2023 LSD outbreak investigation in Bhutan encompassed both active and passive surveillance to identify affected households and animals. A total of 7815 households across 20 Dzongkhags (districts) and 192 Gewogs (groups of villages) were impacted, with 19,907 animals (16,728 cattle and 3179 yaks) affected. The disease led to 2888 deaths, including 1659 cattle and 1229 yaks, resulting in an apparent case fatality rate (CFR) of 9.92% in cattle and 38.66% in yaks. The first case was reported on 2 January 2023 in Samtse district (Geo‐coordinates: 26.9131, 89.0837), and the last case was recorded in Thimphu district (Geo‐coordinates: 27.4721, 89.6380) on 19 September 2023. As illustrated in the spatio‐temporal distribution map (Figure S1), the likely source of the outbreak was suspected to be a cross‐border incursion through the southern entry point, followed by further spread into the interior regions of the country. The outbreaks eased following the initiation of a national LSD vaccination program in August 2023, during which 92.51% of the eligible bovine population in the country were vaccinated using the Neethling vaccine strain of LSDV. Samples for laboratory confirmation and further analysis were collected from clinically affected animals during outbreak investigations.

**Figure 1:**
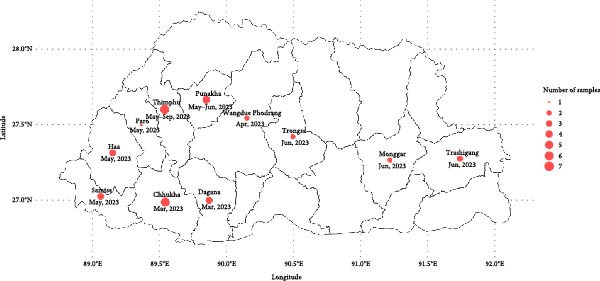
Map of Bhutan showing cases of LSD in cattle and yaks from various districts from which samples were collected for characterization of LSDV.

A total of 42 samples were collected from 12 districts for the characterization of LSDV (Table 1 and Figure 1). Thirty‐six of these samples were from diseased cattle, consisting of 25 whole blood samples, seven tissue samples, one skin scab, two nasal swabs and one serum. The remaining six samples were from yaks, comprising three whole blood samples from diseased yaks and three dried meat samples. The blood samples were collected and shipped in EDTA vacutainers, while the tissue samples were collected in dry tubes. The samples were transported from the field to the laboratory in a cool box with ice packs for the initial analysis. The three dried yak meat samples were received at the laboratory in a plastic container.

**Table 1:** Description of the LSD outbreak in bovine populations across the districts in Bhutan, including the sample types collected for LSDV characterization.

District	No. of susceptible animals	Bovine species affected	No. of lethal cases	No. of live clinical cases	Type of sample collected	Animal species		Collection date	Sample ID	Total no. of samples
						Cattle	Yak			
Chhukha	16588	Cattle	46	561	Blood	2	—	Mar‐23	Bhu 1,2	7
					Skin scraping	1			Bhu 3	
					Serum	1			Bhu 8	
					Tissue	3			Bhu 9,10,11	
Dagana	15720	Cattle	59	988	Tissue	2	—	Mar‐23	Bhu 4, 7	4
					Nasal swab	2			Bhu 5, 6	
Haa	8927	Cattle and yak	472	813	Blood	4	—	May‐23	Bhu 12,13, 18, 19	4
Monggar	26302	Cattle	178	1704	Blood	2	—	Jun‐23	Bhu 41, 42	2
Paro	13137	Cattle and yak	237	1554	Blood	1	—	May‐23	Bhu 20	1
Pema Gatsel	7198	Cattle	36	576	Blood	1	—	Apr‐23	Bhu 21	1
Punakha	9670	Cattle	37	801	Blood	5	—	May‐23	Bhu 24,25,26,27	5
								Jun‐23	Bhu 36	
Samtse	33474	Cattle	69	1076	Blood	4	—	May‐23	Bhu 14,15,16,17	4
Thimphu	12234	Cattle and yak	764	1982	Blood	2	—	May‐23	Bhu 29,39	7
					Blood	—	2	Aug‐23	Bhu 30,31	
					Dried meat	—	3	Sep‐23	Bhu 33,34,35	
Trashigang	35717	Cattle and yak	343	944	Blood	2	—	Jun‐23	Bhu 37, 40	3
					Blood	—	1	Jun‐23	Bhu 32	
Trongsa	8170	Cattle	13	501	Tissues	2	—	Jun‐23	Bhu 28	2
									Bhu 38	
Wangdue Phodrang	22914	Cattle and yak	89	624	Blood	2	—	Apr‐23	Bhu 22, 23	2
Bumthang	13997	Cattle and yak	41	140	N/A	N/A	N/A	N/A	N/A	N/A
Gasa	6365	Cattle	9	81	N/A	N/A	N/A	N/A	N/A	N/A
Lhuentse	12500	Cattle	38	442	N/A	N/A	N/A	N/A	N/A	N/A
Samdrup Jongkhar	12133	Cattle	59	577	N/A	N/A	N/A	N/A	N/A	N/A
Sarpang	16578	Cattle	135	997	N/A	N/A	N/A	N/A	N/A	N/A
Trashi Yangtse	9846	Cattle	12	312	N/A	N/A	N/A	N/A	N/A	N/A
Tsirang	12193	Cattle	238	2036	N/A	N/A	N/A	N/A	N/A	N/A
Zhemgang	10085	Cattle	13	13	N/A	N/A	N/A	N/A	N/A	N/A
Total	303748		2888	17019		36	6			42

### 2.2. Sample Processing and Molecular Detection of *Capripoxvirus* Genome

Approximately, 25 mg of tissue was homogenized in PBS, clarified by centrifugation, and the supernatant was collected. 200 µL of the supernatant or 200 µL of blood samples were used for DNA extraction using the QIAamp DNA mini kit following the manufacturer’s instructions, and the DNA was eluted in 200 µL of nuclease‐free water.

The extracted DNA samples were screened for the presence of *capripoxvirus* (CaPV) DNA using a qPCR method previously described by Bowden et al. [10]. Briefly, the PCR reactions were prepared in a 20 *μ* L reaction volume containing 18 *μ* L of the PCR master mix, 400 nM of each of the forward and reverse primers, 250 nM of the probe, and 2 *μ* L of template DNA. The real‐time PCR program consisted of an initial denaturation at 95°C for 10 min, followed by 45 cycles at 95°C for 15 s and at 60°C for 60 s.

### 2.3. Molecular Characterization of LSDV Based on Selected CaPV Genes

For all CaPV‐positive samples, the complete RNA polymerase 30 kDa subunit (RPO30), G protein‐coupled receptor (GPCR) genes, the partial extracellular enveloped virus (EEV) glycoprotein, and the CaPV homolog of the variola virus B22R genes were amplified and sequenced as previously described [11–13]. Collectively, these targeted genes enable the differentiation of LSDV field strains from vaccines and known recombinants. Following the analysis of PCR products on a 2% agarose gel in 1×TAE buffer and visualization on a Gel Documentation System (Bio‐Rad), the samples were purified and sent for Sanger sequencing at LGC Genomics (Germany).

The obtained raw sequences were assembled using Vector NTI software v11.5 (Invitrogen). In the subsequent comparative analyses, only samples for which all the CaPV‐targeted genes were successfully sequenced were included. The sequences of each targeted gene were aligned with their homologous sequences from other LSDVs, SPVs, and GPVs retrieved from GenBank, using the Muscle algorithm with the codon option in MEGA X [14]. Neighbor‐joining trees of the complete RPO30 and GPCR genes were then constructed using the same software. The evolutionary distances were computed using the maximum composite likelihood method with 1000 bootstrap replicates. The trees were visualized with the Interactive Tree of Life (iTOL) tool [15], together with the associated metadata. The insertion/deletions in the GPCR or the EEV glycoprotein associated with LSDV clades were visualized with the RPO30 tree. For the partial EEV glycoprotein and B22R genes, multiple sequence alignments and visualization were done using BioEdit v7.2.5.

### 2.4. Whole Genome Sequencing and Analysis

Whole genome sequencing of the skin scraping sample LSDV_Bhutan_03, collected from cattle, was performed using the Pacific Biosciences (PacBio) Sequel IIe platform. For library construction, 300 ng of DNA was used. Following quality and size distribution assessment using Agilent Genomic DNA ScreenTape on Tapestation 2200, the SMRTbell libraries were prepared with the SMRTbell prep kit 3.0, which involves shearing, repair, A‐tailing, ligation with barcodes, and adapters, and size selection with SMRTbell clean‐up beads. Pooled libraries ( ≥ 300 ng) were converted to SMRTbell libraries and sequenced on the Sequel IIe (PacBio) platform under CCS mode for 30 h.

The reads were classified using the Centrifuge classifier. The LSDV‐matching reads were then extracted and assembled de novo using Canu v2.3, Flye v2.2.2, and Unicycler v0.50. Alternatively, de novo assembly was performed directly with Improved Phased Assembler (IPA) without host removal or with Canu after removing cattle reads. Assemblies were compared, and any discrepancies were assessed through mapping back the reads to de novo contigs using minimap2 v2.26 and visualization with IGV. Variants were analyzed using bcftools. Consensus sequences were extracted with bcftools, seqtk, and vcfutils, and coverage was determined using qualimap v2.3.

The open reading frames (ORFs) of the LSDV Bhutan genome were predicted using GATU [16], with LSDV_NW‐LW (AF409137) as a reference genome. The complete genome of LSDV_Bhutan_03 was submitted to GenBank under the Accession Number PQ878211. To compare the LSDV Bhutan genome with publicly available LSDV genomes, a dataset with 80 LSDV whole genome sequences was aligned using MAFFT [17]. A median‐joining network was then constructed using the PopART program, with epsilon set to zero and mutations shown as numbers [18].

## 3. Results and Discussion

### 3.1. LSD Outbreak Investigation and Clinical Signs

**Figure 2:**
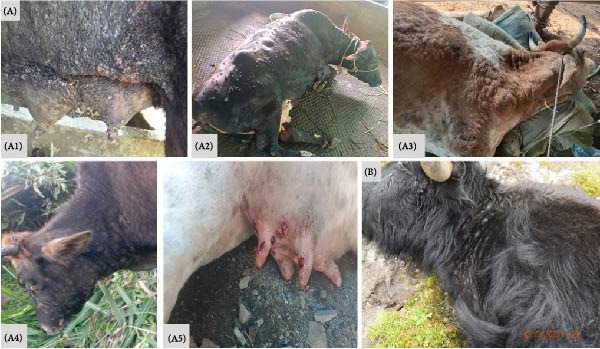
Clinical signs of LSD, (A) in cattle, with lesions spread all over the body (A1, A2, and A3), the neck (A4) and udder (A5), and (B) in yak.

The first confirmed case of LSD in Bhutan was in October 2020, which was initially contained within the southern districts of the country. However, a significant surge of LSD cases was observed following the easing of COVID‐19 restrictions, and by the summer season of 2023, the disease had spread to all districts, affecting both cattle and yaks. The progression of the outbreak appeared to correlate with rising temperatures, which likely facilitated the northward transmission and cross‐species spread from cattle to yaks. This transmission pattern is consistent with findings from other regions, where vectors such as biting flies, along with factors like animal trade and human movement, have significantly contributed to the dissemination of LSD [19–21]. In cattle, the clinical symptoms of LSD observed were fever, lameness, diarrhea, salivation, loss of milk production, and nodules and wounds on the skin, including the head, neck, udder, scrotum, perineum, and buccal mucosa (Figure 2A) [2]. In yaks, the symptoms included high fever, loss of appetite (anorexia), weakness, nasal discharge, excessive salivation, reduction in milk yield, nodules and wounds on the skin, swollen limbs and abdomen, and in some cases death and abortion (Figure 2B). These typical LSD clinical signs were consistent with those reported in yaks from Tibet, China, and Sikkim, India, highlighting the severity and impact of the disease [22, 23].

In Bhutan, the CFR for LSD was recorded at 9.92% in cattle and 38.66% in yaks. Typically, LSD‐affected areas report CFRs ranging from 3.3% to 23.9% [24–26]; however, cases with CFRs as high as 50% have been documented [27]. The high CFR observed in yaks in Bhutan is comparable to reports from China and India, where LSD has similarly led to significant mortality rates in yak populations [23, 27–29]. Although the clinical presentation in yaks resembled that in cattle, the disease course in yaks was notably more severe, resulting in a higher CFR. Several factors may have contributed to this outcome. First, yak populations undergo seasonal migration to high‐altitude summer pastures, a process associated with considerable physiological stress that may compromise immune function [30–32]. Second, the initial LSD outbreak in Bhutan was reported in Samtse district, bordering West Bengal, India. That outbreak was swiftly contained, and no further spread was documented at the time. It is possible that cattle in Samtse developed some level of natural immunity due to prior exposure, although this has not been confirmed through serological testing. In contrast, cattle in other districts and yak populations had no history of exposure to LSDV and were immunologically naïve. Therefore, the lack of prior exposure or vaccination, combined with physiological stress, could have contributed to the high mortality observed in yaks [33–35]. These findings highlight the urgent need for timely vaccination and improved disease surveillance to mitigate the impact of LSD in vulnerable livestock populations.

### 3.2. Molecular Detection of LSDV

Diagnosis of LSD based on clinical signs can be confused with other diseases such as pseudo LSD, bovine dermatophilosis, onchocerciasis, besnoitiosis, demodex infection, insect bite, urticaria, and photosensitization [36–38]. Therefore, RT‐PCR described by Bowden et al. [10] was employed for accurate diagnosis of the LSD cases. CaPV genome was detected in 34 out of 42 samples (81%) using the RT‐PCR. Of these, 29 were cattle samples, and five were yak samples (3 blood and 2 dried meat samples) (Table S1).

### 3.3. Sequence and Phylogenetic Analysis of the Targeted CaPV Genes

For further analysis, 16 CaPV‐positive samples with a cycle threshold (Ct) value ≤ 30 were chosen and the RPO30, GPCR, EEV, and B22R genes were amplified and sent for sequencing. All targeted genes were successfully sequenced for eight of the 16 samples and further analyzed. The sequences generated for each gene were deposited in GenBank with accession numbers PQ858710‐PQ858741. The multiple sequence alignments showed 100% similarity among the eight LSDV‐positive samples from Bhutan for all four targeted genes (RPO30, GPCR, EEV, and B22R), including the two yak samples. The genetic similarities among the samples suggest a possible spillover of the virus between cattle and yaks, as seen in other regions such as India and China [22, 39].

**Figure 3:**
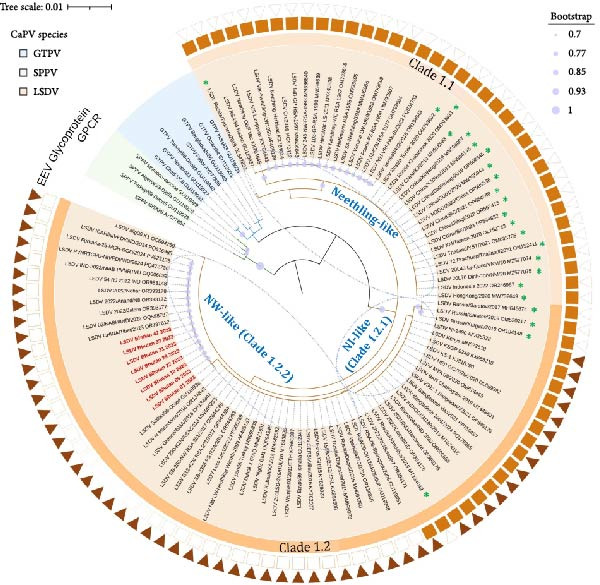
Neighbor‐joining tree based on the complete RPO30 gene sequences of CaPVs with LSDVs from Bhutan (in red) visualized on iTOL, along with LSDV clustering based on the presence (filled box) or absence (empty box) of sequence insertion in the GPCR and the EEV glycoprotein genes. The evolutionary distances were computed using the Tamura‐Nei method, with 1000 bootstrap replicates on MEGA X. Recombinant LSDVs are marked with an asterisk.

Phylogenetic analysis based on the complete RPO30 gene sequences indicated that Bhutan LSDV isolates from cattle and yaks clustered within LSDV Clade 1.2, which includes the commonly circulating LSDV field isolates from Africa, Asia, the Middle East, and Europe [40]. Within Clade 1.2, the Bhutan LSDV isolates grouped with Neethling Warmbaths (NW)‐like LSDVs classified under Clade 1.2.2, such as isolates from cattle in Obied, Sudan (GU119938_LSDV Sudan/06‐Obied_2006) and Kashmir, India (OQ588787_LSDV/02/KASH/IND/2022), as well as in cattle–yak hybrids in Tibet, China (OR797612_LSDV/CHINA/Tibet/2023) and buffalo in India (PVNRTGVU‐NIVEDI/2024/0608) (Figure 3) [23, 41, 42]. Similarly, phylogenetic analysis based on the GPCR gene grouped the LSDVs from Bhutan within LSDV Clade 1.2 (Figure S2). Additionally, the GPCR sequence alignment revealed a 12‐nucleotide deletion in LSDVs from Bhutan, similar to common LSDV field isolates, NW‐like LSDVs, which distinguishes these isolates from NI‐like LSDVs in the same clade, as well as from the Neethling‐like and recombinant LSDVs (Figure 3) [43]. Furthermore, multiple sequence alignments of the partial EEV glycoprotein gene demonstrated that the LSDVs from Bhutan were also similar to common LSDV field isolates, as evidenced by a 27‐nucleotide insertion (Positions 175–201). This insertion reiterated that the viruses from Bhutan were distinct from Neethling‐like LSDVs and the recombinant LSDVs from South‐East Asia and Russia (Figure 3, Figure S3) [44].

**Figure 4:**
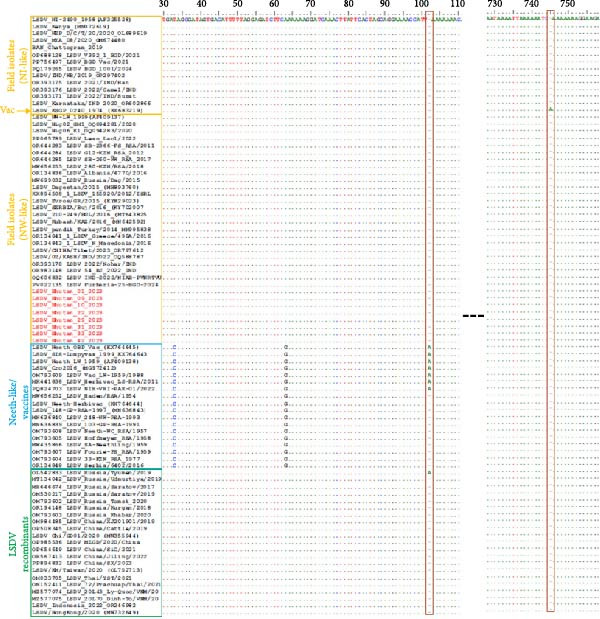
Multiple sequence alignment of the partial nucleotide sequences of the B22R gene. The sequences from the LSDVs from Bhutan (in red) were aligned with representative LSDV sequences retrieved from GenBank. The nucleotide insertion in LSDV Neethling and LSDV KSGPO‐240 vaccines, which are absent in the viruses from Bhutan, are shown in the red highlighted columns. The dots indicate the identical nucleotides in the alignment.

Moreover, the B22R sequences of the LSDVs from Bhutan resembled the conventional LSDV field isolates (NW‐ and NI‐like LSDVs) encountered in Africa, Europe, and Asia. However, they differed from LSDV Neethling and LSDV KSGP‐0240 derived vaccines, which exhibit a nucleotide insertion at positions 102 and 745, respectively (Figure 4) [45].

### 3.4. Bhutan LSDV Whole Genome Analysis

Read classification of the LSDV_Bhutan_03 sample revealed the presence of multiple microbial pathogens, including 45,467 reads of *Mannheimia varigena*, 2510 reads of other *Mannheimia* species, 16,062 reads of *Clostridium botulinum*, and 1383 reads of LSDV (Figure S4). Although this sample originated from cattle, the detection of *M. varigena*—a known agent of shipping fever [46] suggests that similar co‐infection combined with the physiological stress of seasonal migration of yaks may have exacerbated disease severity.

The LSDV_Bhutan_03 genome was assembled from 1383 LSDV‐specific reads (0.16%), with sequence lengths ranging from 1094 to 14,404 bp (mean length: 5562 bp). The final assembled genome was 150,877 bp long, with a mean depth of 50× (min 30× and max 100×), ensuring comprehensive genome coverage. A nucleotide BLAST search of the assembled whole genome showed that over the aligned genome fragment, the LSDV from Bhutan has high similarity (99.93%–100%) to conventional LSDV field isolates such as LSDV/2022/Jalore (OR393177), LSDV/2022/Anand/N9 (OR393173), LSDV/2022/Nohar (OR393178), and LSDV/02/KASH/IND/2022 (OQ588787) found in India in 2022, LSDV/CHINA/Tibet/2023 (OR797612) isolated in China in 2023, Evros/GR/15 (KY829023), and NW‐LW like isolates; such as NW‐LW (AF409137).

**Figure 5:**
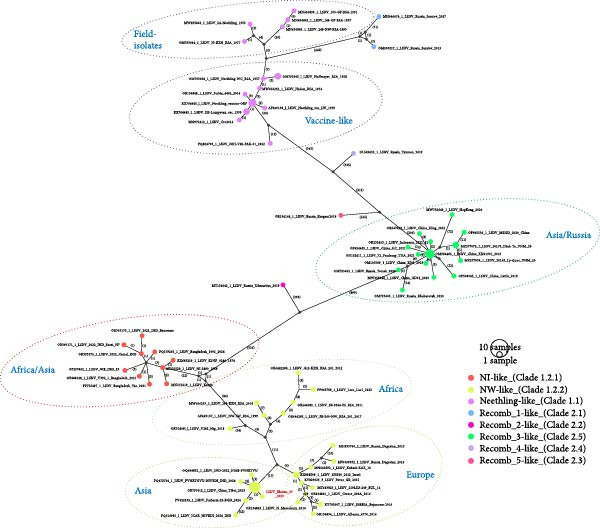
Median‐joining network inferred from LSDV whole‐genome sequences using the PopART program. The network shows the LSDV genome from Bhutan (in red) clustering with NW‐like LSDVs, and closer to some of the LSDVs isolates from India, Bangladesh, and China. The number of mutations between each genome is labelled.

The LSDV_Bhutan_03 whole genome consisted of 157 predicted ORFs, with 140 sharing 100% homology to the reference strain, LSDV_NW‐LW isolate (AF409137). Of the remaining 17 ORFs, 14 exhibited 95.9%–99.9% homology with 1–2 single‐nucleotide polymorphisms (SNPs), while 3; LD019b (kelch‐like protein), LD026a (hypothetical protein) and LD087 (mutT motif protein) had lower homology of 85.2%, 71.2%, and 34.8%, respectively, due to indels causing frameshifts and truncation. Notably, LD087, known to downregulate host gene transcription and enhance innate immune response [47], was truncated in the Bhutan isolate and in other recent NW‐LW like LSDV strains circulating in Asia. This truncation may contribute to impaired host immune response and potentially increased virulence and high CFR observed in yaks. Median Joining network analysis, based on 80 aligned LSDV whole genomes using PopART software showed that the LSDV genome from Bhutan clusters together with other viruses in the NW‐like LSDVs cluster (Clade 1.2.2). Within the NW‐like clade, the LSDV from Bhutan was more closely related to the recent LSDVs from India (2022 and 2024), Bangladesh (2024), and China (2023), differing by only one to three synonymous SNPs. In contrast, there were 23 and 36 SNPs between the LSDV from Bhutan and Evros/GR/15 (KY829023) and NW‐LW (AF409137), respectively (Figure 5). Notably, until 2022, only NI‐like LSDV isolates (Clade 1.2.1) were reported in South Asian countries such as India, Bangladesh, Myanmar, and Nepal, as well as recombinant LSDVs (Clade 2.5) circulating in Southeast and Eastern Asia (China, Thailand, Vietnam, Indonesia, and Taiwan) [48]. This indicates that Bhutan is one of the Asian countries, alongside India, Bangladesh, and China, where NW‐like LSDV is circulating.

## 4. Conclusions

These findings underscore the importance of continuous monitoring, accurate diagnosis, and comprehensive genomic analysis to understand the molecular epidemiology of the disease. This is crucial for informing vaccine development, strengthening LSD outbreak preparedness, and guiding effective disease management policies. The observed spread of the disease from southern to northern districts and from cattle to yaks appeared to be influenced by rising temperatures, suggesting vector‐borne transmission may have contributed to the disease transmission. Given that the disease is vector‐borne, further studies on the vectors, including biting flies and their control, could supplement prevention and control programs for LSD. However, this study did not investigate the specific factors responsible for spreading the disease. Therefore, the situation calls for the implementation of stronger biosecurity measures to mitigate the risk of further spread.

## Conflicts of Interest

The authors declare no conflicts of interest.

## Funding

This research was supported by the VETLAB network initiative of the Joint FAO/IAEA Centre, funded through the Peaceful Uses Initiatives (PUI) by Japan and the United States of America.

## Supporting Information

Additional supporting information can be found online in the Supporting Information section.

## Supporting information


**Supporting Information** Figure S1: Map of Bhutan showing spatio‐temporal distribution of LSD outbreaks in the country in 2023. Figure S2: Neighbor‐joining tree based on the complete GPCR gene sequences of CaPVs, with LSDVs from Bhutan (in red), visualized on iTOL. The Tamura‐Nei model Gamma distribution was used. Recombinant LSDVs are marked with an asterisk. Figure S3: Multiple sequence alignment of the partial nucleotide sequences of the EEV glycoprotein gene. The sequences of the LSDVs from Bhutan (in red) were aligned with representative LSDV sequences retrieved from GenBank. A 27‐nucleotide deletion absent in the viruses from Bhutan is highlighted in the box. The dots indicate the identical nucleotides in the alignment. Figure S4: Donut plot illustrating the taxonomic classification of the most abundant pathogen‐associated reads identified in the LSDV_Bhutan_03 sample. Table S1: PCR results of the samples collected from LSD‐suspected cattle and yak cases from Bhutan in 2023. Samples that were successfully sequenced are highlighted in green.

## Data Availability

DNA sequences generated and analyzed in the current study are available in GenBank under Accession Numbers PQ858710 – PQ858741 and PQ878211.
