# Supplementary material for: Molecular Characterization and Epidemiology of Lumpy Skin Disease Virus in Bhutan, 2023
Source: Transbound Emerg Dis. 2025 Dec 11;2025:8634585. doi: 10.1155/tbed/8634585 (PMC12697798; doi:10.1155/tbed/8634585)
Supplement: Supplementary file 1 — Supporting Information Figure S1: Map of Bhutan showing spatio‐temporal distribution of LSD outbreaks in the country in 2023. Figure S2: Neighbor‐joining tree based on the complete GPCR gene sequences of CaPVs, with LSDVs from Bhutan (in red), visualized on iTOL. The Tamura‐Nei model Gamma distribution was used. Recombinant LSDVs are marked with an asterisk. Figure S3: Multiple sequence alignment of the partial nucleotide sequences of the EEV glycoprotein gene. The sequences of the LSDVs from Bhutan (in red) were aligned with representative LSDV sequences retrieved from GenBank. A 27‐nucleotide deletion absent in the viruses from Bhutan is highlighted in the box. The dots indicate the identical nucleotides in the alignment. Figure S4: Donut plot illustrating the taxonomic classification of the most abundant pathogen‐associated reads identified in the LSDV_Bhutan_03 sample. Table S1: PCR results of the samples collected from LSD‐suspected cattle and yak cases from Bhutan in 2023. Samples that were successfully sequenced are highlighted in green. [file TBED-2025-8634585-s001.zip › Supplementary Figure S3.pdf]

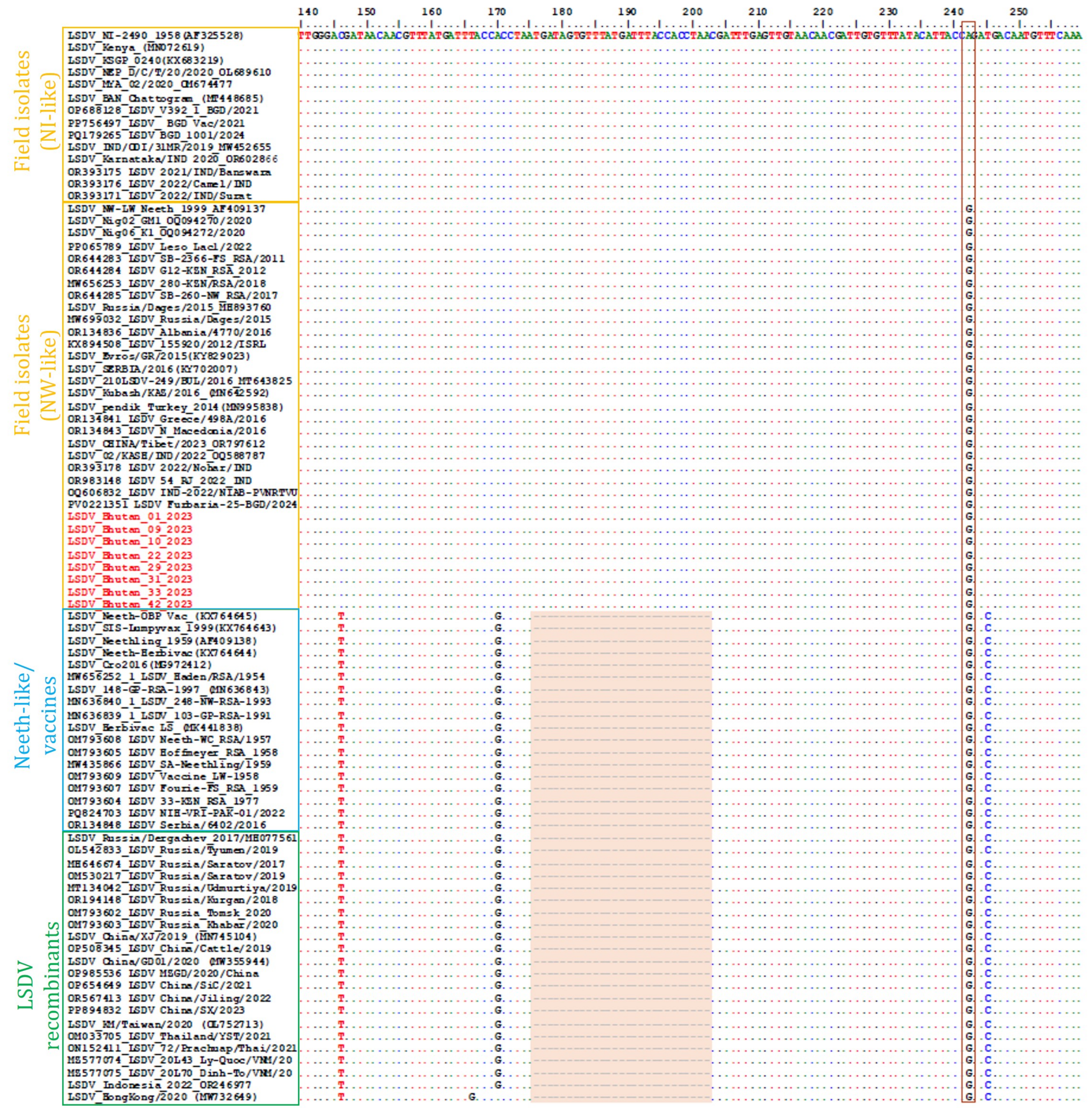

140 150 160 170 180 190 200 210 220 230 240 250
Field isolates (NI-like)
LSDV_NI-2490_1958(AF325528)
TTGGACGATAACAACGTTTATGATTTACCACCTAATGATAGTGTTTATGATTTACCACCTAACGATTTGAGTTGTAACAACGATTGTGTTTATACATTACCAGATGACAATGTTTCAAA
LSDV_Kenya_(MN072619)
LSDV_KSGP_0240(KX683219)
LSDV_NEP_D/C/T/20/2020_OL689610
LSDV_MYA_02/2020_OM674477
LSDV_BAN_Chattogram_(MT448685)
OP688128_LSDV_V392_I_BGD/2021
PP756497_LSDV_ BGD Vac/2021
PQ179265_LSDV_BGD_1001/2024
LSDV_IND/ODI/31MR/2019_MW452655
LSDV_Karnataka/IND 2020_OR602866
OR393175_LSDV_2021/IND/Banswara
OR393176_LSDV_2022/Camel/IND
OR393171_LSDV_2022/IND/Surat
Field isolates (NW-like)
LSDV_NW-LW_Neeth_1999_AF409137
LSDV_Nig02_GM1_OQ094270/2020
LSDV_Nig06_K1_OQ094272/2020
PP065789_LSDV_Leso_Lac1/2022
OR644283_LSDV_SB-2366-FS_RSA/2011
OR644284_LSDV_G12-KEN_RSA_2012
MW656253_LSDV_280-KEN/RSA/2018
OR644285_LSDV_SB-260-NW_RSA/2017
LSDV_Russia/Dages/2015_MH893760
MW699032_LSDV_Russia/Dages/2015
OR134836_LSDV_Albania/4770/2016
KX894508_LSDV_155920/2012/ISRL
LSDV_Evros/GR/2015(KY829023)
LSDV_SERBIA/2016(KY702007)
LSDV_210LSDV-249/BUL/2016_MT643825
LSDV_Kubash/KAZ/2016_(MN642592)
LSDV_pendik_Turkey_2014(MN995838)
OR134841_LSDV_Greece/498A/2016
OR134843_LSDV_N_Macedonia/2016
LSDV_CHINA/Tibet/2023_OR797612
LSDV_02/KASH/IND/2022_OQ588787
OR393178_LSDV_2022/Nohar/IND
OR983148_LSDV_54_RJ_2022_IND
OQ606832_LSDV_IND-2022/NIAB-PVNRTVU
PV022135I_LSDV_Furbaria-25-BGD/2024
LSDV_Bhutan_01_2023
LSDV_Bhutan_09_2023
LSDV_Bhutan_10_2023
LSDV_Bhutan_22_2023
LSDV_Bhutan_29_2023
LSDV_Bhutan_31_2023
LSDV_Bhutan_33_2023
LSDV_Bhutan_42_2023
Neeth-like/ vaccines
LSDV_Neeth-OBP_Vac_(KX764645)
LSDV_SIS-Lumpyvax_1999(KX764643)
LSDV_Neethling_1959(AF409138)
LSDV_Neeth-Herbivac(KX764644)
LSDV_Cro2016(MG972412)
MW656252_1_LSDV_Haden/RSA/1954
LSDV_148-GP-RSA-1997_(MN636843)
MN636840_1_LSDV_248-NW-RSA-1993
MN636839_1_LSDV_103-GP-RSA-1991
LSDV_Herbivac_LS_(MK441838)
OM793608_LSDV_Neeth-WC_RSA/1957
OM793605_LSDV_Hoffmeyer_RSA_1958
MW435866_LSDV_SA-Neethling/1959
OM793609_LSDV_Vaccine_LW-1958
OM793607_LSDV_Fourie-FS_RSA_1959
OM793604_LSDV_33-KEN_RSA_1977
PQ824703_LSDV_NIH-VRI-PAK-01/2022
OR134848_LSDV_Serbia/6402/2016
LSDV recombinants
LSDV_Russia/Dergachev_2017/MH077561
OL542833_LSDV_Russia/Tyumen/2019
MH646674_LSDV_Russia/Saratov/2017
OM530217_LSDV_Russia/Saratov/2019
MT134042_LSDV_Russia/Udmurtiya/2019
OR194148_LSDV_Russia/Kurgan/2018
OM793602_LSDV_Russia_Tomsk_2020
OM793603_LSDV_Russia_Khabar/2020
LSDV_China/XJ/2019_(MN745104)
OP508345_LSDV_China/Cattle/2019
LSDV_China/GD01/2020_(MW355944)
OP985536_LSDV_MEGD/2020/China
OP654649_LSDV_China/SiC/2021
OR567413_LSDV_China/Jiling/2022
PP894832_LSDV_China/SX/2023
LSDV_KM/Taiwan/2020_(OL752713)
OM033705_LSDV_Thailand/YST/2021
ON152411_LSDV_72/Prachuap/Thai/2021
MS577074_LSDV_20L43_Ly-Quoc/VNM/20
MS577075_LSDV_20L70_Dinh-To/VNM/20
LSDV_Indonesia_2022_OR246977
LSDV_HongKong/2020_(MW732649)
