# Supplementary figures and images for: Molecular Characterization and Epidemiology of Lumpy Skin Disease Virus in Bhutan, 2023
Source: Transbound Emerg Dis. 2025 Dec 11;2025:8634585. doi: 10.1155/tbed/8634585 (PMC12697798; doi:10.1155/tbed/8634585)

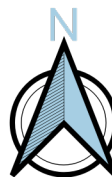

Outbreak Month

- Jan - April
- May - June
- July

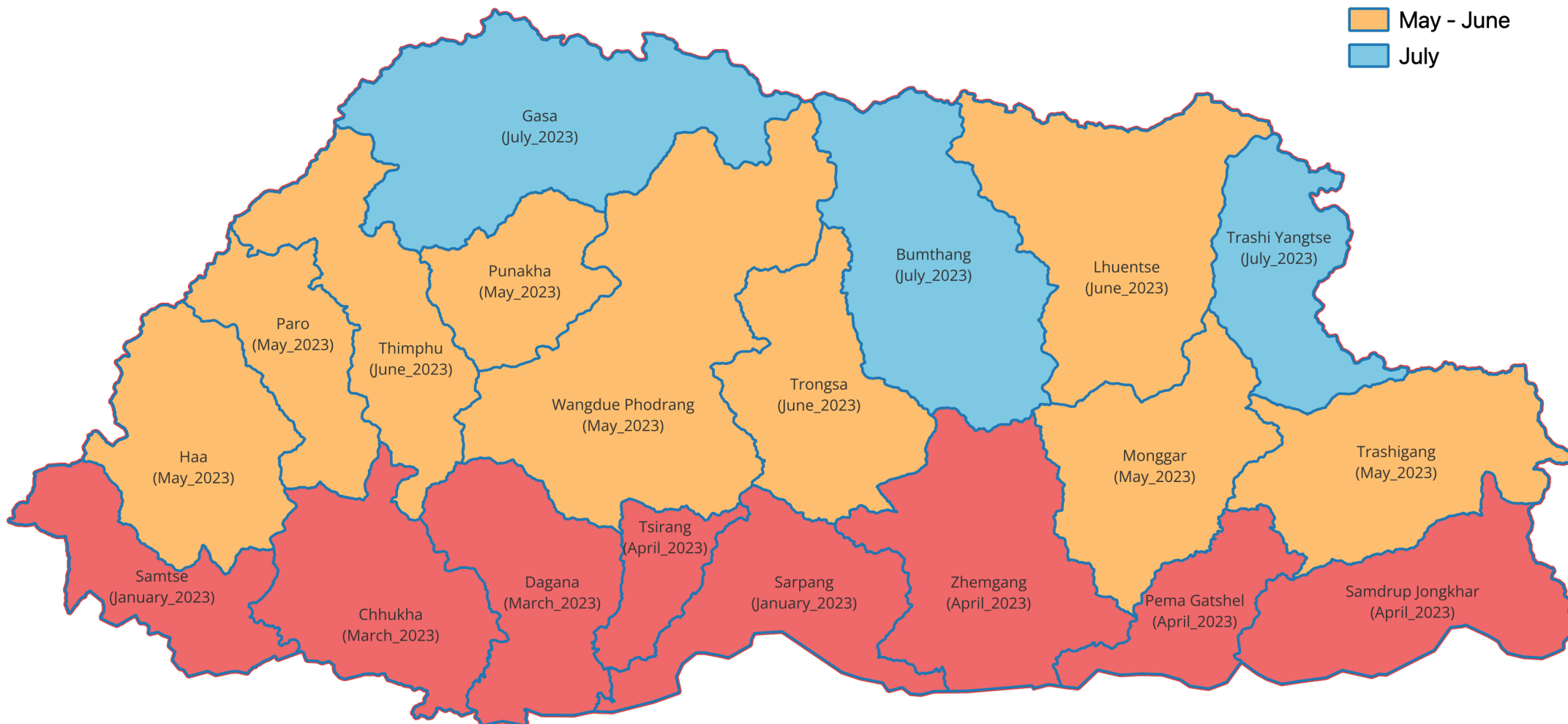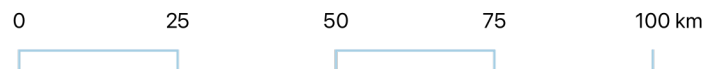

Supplement: Supplementary file 1 — Supporting Information Figure S1: Map of Bhutan showing spatio‐temporal distribution of LSD outbreaks in the country in 2023. Figure S2: Neighbor‐joining tree based on the complete GPCR gene sequences of CaPVs, with LSDVs from Bhutan (in red), visualized on iTOL. The Tamura‐Nei model Gamma distribution was used. Recombinant LSDVs are marked with an asterisk. Figure S3: Multiple sequence alignment of the partial nucleotide sequences of the EEV glycoprotein gene. The sequences of the LSDVs from Bhutan (in red) were aligned with representative LSDV sequences retrieved from GenBank. A 27‐nucleotide deletion absent in the viruses from Bhutan is highlighted in the box. The dots indicate the identical nucleotides in the alignment. Figure S4: Donut plot illustrating the taxonomic classification of the most abundant pathogen‐associated reads identified in the LSDV_Bhutan_03 sample. Table S1: PCR results of the samples collected from LSD‐suspected cattle and yak cases from Bhutan in 2023. Samples that were successfully sequenced are highlighted in green. [file TBED-2025-8634585-s001.zip › Supplementary Figure S1.pdf]

### CaPV species

 GTPV

 SPPV

 LSDV

bootstrap

- 0.7

0.77

0.85

0.93

1

### Clade 1.1

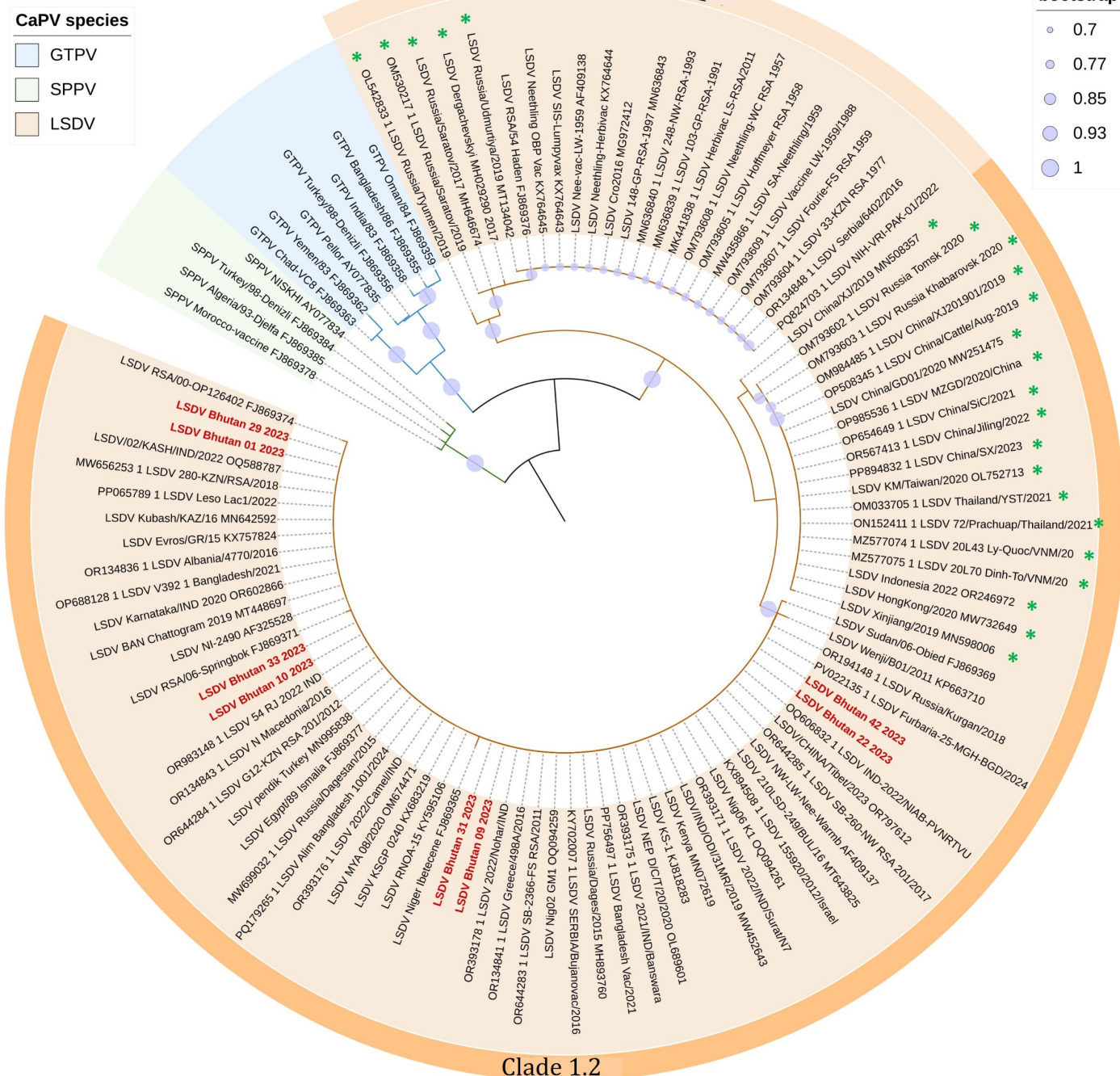

## Clade 1.2

Supplement: Supplementary file 1 — Supporting Information Figure S1: Map of Bhutan showing spatio‐temporal distribution of LSD outbreaks in the country in 2023. Figure S2: Neighbor‐joining tree based on the complete GPCR gene sequences of CaPVs, with LSDVs from Bhutan (in red), visualized on iTOL. The Tamura‐Nei model Gamma distribution was used. Recombinant LSDVs are marked with an asterisk. Figure S3: Multiple sequence alignment of the partial nucleotide sequences of the EEV glycoprotein gene. The sequences of the LSDVs from Bhutan (in red) were aligned with representative LSDV sequences retrieved from GenBank. A 27‐nucleotide deletion absent in the viruses from Bhutan is highlighted in the box. The dots indicate the identical nucleotides in the alignment. Figure S4: Donut plot illustrating the taxonomic classification of the most abundant pathogen‐associated reads identified in the LSDV_Bhutan_03 sample. Table S1: PCR results of the samples collected from LSD‐suspected cattle and yak cases from Bhutan in 2023. Samples that were successfully sequenced are highlighted in green. [file TBED-2025-8634585-s001.zip › Supplementary Figure S2.pdf]

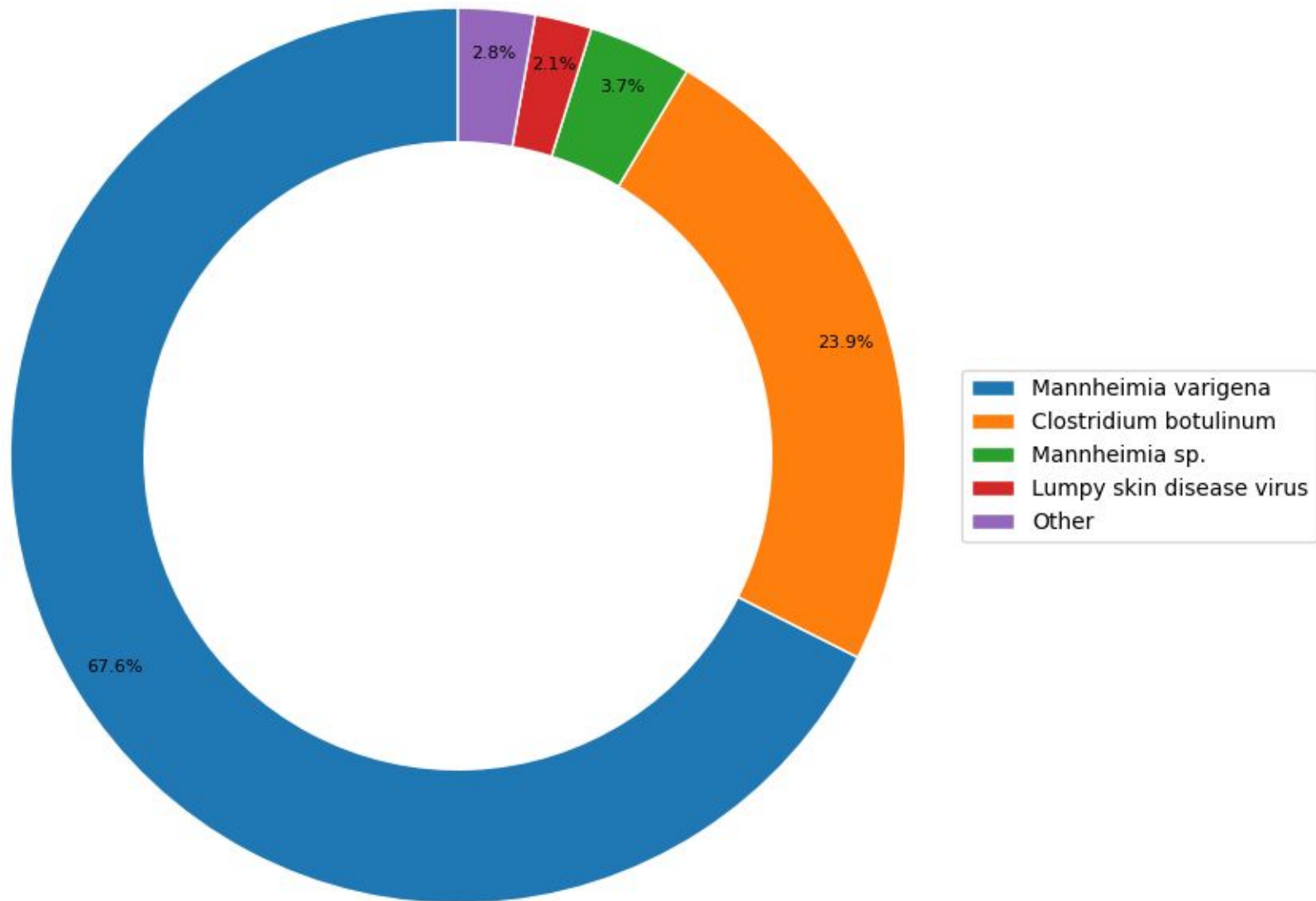

Supplement: Supplementary file 1 — Supporting Information Figure S1: Map of Bhutan showing spatio‐temporal distribution of LSD outbreaks in the country in 2023. Figure S2: Neighbor‐joining tree based on the complete GPCR gene sequences of CaPVs, with LSDVs from Bhutan (in red), visualized on iTOL. The Tamura‐Nei model Gamma distribution was used. Recombinant LSDVs are marked with an asterisk. Figure S3: Multiple sequence alignment of the partial nucleotide sequences of the EEV glycoprotein gene. The sequences of the LSDVs from Bhutan (in red) were aligned with representative LSDV sequences retrieved from GenBank. A 27‐nucleotide deletion absent in the viruses from Bhutan is highlighted in the box. The dots indicate the identical nucleotides in the alignment. Figure S4: Donut plot illustrating the taxonomic classification of the most abundant pathogen‐associated reads identified in the LSDV_Bhutan_03 sample. Table S1: PCR results of the samples collected from LSD‐suspected cattle and yak cases from Bhutan in 2023. Samples that were successfully sequenced are highlighted in green. [file TBED-2025-8634585-s001.zip › Supplementary Figure S4.pdf]
